# Efficacy of dexamethasone or clonidine as adjuvants in interscalene brachial plexus block for preventing rebound pain after shoulder surgery: a randomized clinical trial

**DOI:** 10.1016/j.bjane.2024.844575

**Published:** 2024-11-24

**Authors:** Layana Vieira Nobre, Leonardo Henrique Cunha Ferraro, Juscelino Afonso de Oliveira Júnior, Vitória Luiza Locatelli Winkeler, Luis Flávio França Vinhosa Muniz, Hiago Parreão Braga, Plínio da Cunha Leal

**Affiliations:** a Hospital Geral de Fortaleza (HGF), Departamento de Anestesiologia, Fortaleza, CE, Brazil; b Universidade Federal de São Paulo (UNIFESP), Departamento de Anestesiologia, São Paulo, SP, Brazil; c Hospital Universitário Cajuru, Departamento de Anestesiologia, Curitiba, PR, Brazil; d Universidade Federal de Goiás (UFG), Departamento de Anestesiologia, Goiânia, GO, Brazil; e Hospital São Domingos, Departamento de Anestesiologia, São Luis, MA, Brazil; f Universidade Federal do Maranhão (UFMA), Programa de Pós-Graduação em Saúde do Adulto, São Luis, MA, Brazil

**Keywords:** Brachial plexus block, Clonidine, Dexamethasone, Pain

## Abstract

**Background:**

Rebound pain is believed to involve both nociceptive pain due to insufficient analgesia and hyperalgesia induced by regional anesthesia. Adjuvant's addition could prevent rebound pain. This study aimed to determine if the addition of dexamethasone or clonidine to local anesthetic when performing interscalene block could prevent rebound pain.

**Methods:**

This was a multicenter, prospective, parallel grouping, randomized clinical trial conducted with patients receiving a single injection of bupivacaine 0.375% in interscalene block ultrasound guided and general anesthesia for shoulder surgery were randomly assigned to either no additives (control), clonidine (30 mcg), or dexamethasone (4 mg). The primary outcome was rebound pain, defined as sudden onset of pain, moderate to severe intensity (VAS ≥7) without improvement with oral medication, followed by VAS pain at rest, required rescue analgesia, the occurrence of adverse events or complications, and satisfaction survey assessments between groups. Rebound pain and pain at rest were assessed 2, 4, 6, 12, 24, and 48 hours after the procedure.

**Results:**

The incidence of rebound pain was not statistically different between groups (p-value = 0.22), with an observed incidence of 41.2% (95% CI 25.9‒57.9), 28.6% (95% CI 16.7‒43.3), and 23.3% (95% CI 12.6‒37.6) in the control, dexamethasone, and clonidine groups, respectively. Additionally, there were no significant differences between the groups in time, from anesthetic blockade to first complaint of pain or the severity of postoperative pain.

**Conclusion:**

The administration of dexamethasone or clonidine as perineural adjuncts to local anesthesia in single-injection interscalene blocks did not significantly reduce the incidence of rebound pain.

## Introduction

Interscalene block placed before surgery is widely used for pain management after shoulder procedures.^1^ Peripheral nerve blocks can be associated with many advantages, including early mobilization, shorter hospital stay, adequate pain control, lower incidence of postoperative nausea and vomiting, increased patient satisfaction, and opioid sparing.^2^^,^^3^

However, studies have reported that patients who receive an interscalene block may perceive a more abrupt and disturbing pain trajectory once a block wears off suddenly, often at home, when compared to patients that had no block at all and were managed by conventional analgesics, including high-dose opioids.^4^ This phenomenon, described as rebound pain, may entail a separate manifestation of hyperalgesia above and beyond the block simply 'wearing off', culminating in a sudden increase in postoperative pain (as measured by a Visual Analog Scale – VAS) at the end of the blockade duration (onset 12 to 48 hours after surgical intervention).

There is a paucity of current strategies to prevent or attenuate rebound pain. Studies have suggested perineural dexamethasone to prevent rebound pain,^5^^,^^6^ but this practice may not be risk-free, as long-term safety has not been evaluated.^7^ Prolonging the duration of interscalene blockade can improve patient recovery and comfort. This can be achieved with adjuvants, such as buprenorphine, clonidine, dexamethasone, dexmedetomidine, and magnesium^8^, ^9^, ^10^ or carefully selected adjuvant combinations based on prior in vitro^11^ and in vivo^12^ studies.

Some authors suggest that the mechanism of rebound pain may involve sudden exposure to nociceptive pain due to insufficient preemptive multimodal analgesia, as well as hyperalgesia induced by regional anesthesia and surgical trauma.^13^ Regional anesthesia/local anesthetic-induced hyperalgesia may mechanistically be mediated by heat fibers.^14^ Younger age, female sex, higher preoperative pain score, and bone surgery have also been suggested as risk factors.^15^^,^^16^

It is hypothesized that the occurrence of rebound pain and the consumption of rescue analgesics during the postoperative period of shoulder surgeries can be reduced by combining adjuvants with local anesthetic solutions used to perform brachial plexus blocks.

Our primary aim is to assess whether the addition of adjuvant medications (dexamethasone or clonidine) to local anesthetics while performing a single-injection interscalene block prevents subsequent rebound pain in patients undergoing elective shoulder surgery.

## Methods

### Study design and participants

This was a multicenter, prospective, parallel grouping, randomized clinical trial. Adult patients (aged 18 years or older) with an American Society of Anesthesiologists (ASA) physical status classification of I or II, who underwent shoulder surgery at three tertiary Brazilian hospitals (Hospital Universitário Cajuru – Grupo Marista da Pontifícia Universidade Católica do Paraná, Curitiba, Paraná; Hospital de Urgências de Goiânia, Goiás; and Hospital São Domingos, São Luís, Maranhão) were eligible.

The non-inclusion criteria were the presence of neuropsychiatric diseases, cognitive impairment, or mental status changes; Monoamine Oxidase Inhibitor (MAOI) or anticonvulsant therapy; other fractures, injuries, or previous surgery on the same limb; paresis or paresthesia of the upper limb not attributable to the main diagnosis; and refusal to participate in the study.

### Randomization

Patients were randomly assigned to undergo interscalene block with a single injection of 20 mL bupivacaine 0.375% without vasoconstrictor, without dexamethasone or clonidine (control group); with 30 mcg clonidine (clonidine group); or with 4 mg dexamethasone (dexamethasone group).

An online randomization service generated a random group allocation sequence (Sealed Envelope, London, England). Opaque-sealed envelopes were used to ensure concealment.

Patients, surgeons, anesthesiologists, nurses, data collectors, and outcome assessors were unaware of group allocation.

### Outcomes

The primary outcome of this study was the incidence difference between groups of rebound pain, defined as sudden onset of pain, moderate to severe intensity (VAS ≥7) without improvement with oral medication at any of the evaluated moments.

The secondary outcomes were VAS pain at rest, required rescue analgesia, the occurrence of adverse events or complications, and satisfaction survey (would you undergo interscalene block again?: yes/no).

### Anesthesia

Patients were monitored according to standard ASA recommendations. All received an ultrasound-guided, single-injection interscalene block administered by an experienced anesthesiologist. Patients were sedated with intravenous midazolam and fentanyl and titrated according to their level of anxiety (1–5 mg midazolam and 100 mcg fentanyl).

Each Patient was placed in the supine position with the head turned away from the side to be blocked. A high-frequency linear array transducer (6‒13 MHz) was placed over the interscalene region to identify the brachial plexus in the short-axis view.

Following the brachial plexus block, anesthetic induction was performed with propofol 2 mg.kg^−1^, fentanyl 4 μg.kg^−1^, and cisatracurium 0.15 mg.kg^−1^ (or atracurium 0.5 mg.kg^−1^, or rocuronium 0.6 mg.kg^−1^). Sevoflurane was used for maintenance, with the MAC adjusted to maintain the desired plane of anesthesia. Intraoperatively, 4 mg of ondansetron, 2g of dipyrone, 100 mg of ketoprofen, or 30 mg of ketorolac were administered.

### Measurement and treatment of postoperative pain

In the first hours after surgery, in the anesthesia recovery room, the following supplemental postoperative analgesia protocol was used: intravenous morphine 4 mg every four hours if the pain was above 3 on the VAS.

Before discharge from anesthesia, still in the post-anesthesia recovery room, all patients were instructed on the use of oral medications during the postoperative period, which was started immediately after discharge from the anesthesia recovery room. The following supplemental postoperative analgesia protocol was used: dipyrone 1g, orally, every six hours, ketoprofen 150 mg, orally, every twelve hours and tramadol 50 mg, orally, every eight hours if pain was above 3 on the VAS.

### Assessment of patient's characteristics and perioperative data

The characteristics and perioperative clinical data, including mean age, BMI (Body Mass Index), preoperative pain score, and duration of surgery, were collected from medical records or documented by a team member. Also, VAS, ranging from 0 (no pain) to 10 (worst possible pain) were collected on baseline (pre-block), 30 minutes after block and 30 minutes in PACU (Post-Anesthesia Care Unit).

### Assessment of postoperative pain

Upon PACU discharge and 2, 4, 6, 12 and 24 after PACU discharge, VAS, ranging from 0 (no pain) to 10 (worst possible pain), was quantified and the cumulative dose of rescue opioids was registered and the patient was asked about rebound pain occurrence.

After 48 hours after PACU discharge, the patients were contacted by telephone and asked to report the use of postoperative medications prescribed, their VAS pain at rest, and the occurrence of rebound pain (and, if so, its duration) during the first 48 hours after the procedure. Patients were also asked whether they required rescue analgesia, whether any adverse events or complications occurred, and the satisfaction regarding the anesthetic technique, evaluated through a dichotomous question: “Would you undergo interscalene block again?” (yes/no).

### Sample size

The sample size was calculated based on the results obtained by Woo et al.^6^ To compare the mean difference (increase) in pain score before and after interscalene block resolution as assessed by an NRS, considering a mean (standard deviation) score of 4.5±2.4 in the dexamethasone group and 6.9±2.2 in the control group, the minimum sample size was estimated as 25 cases per group. Considering the incidence of rebound pain identified in the aforementioned study (82.9% in the control group and 37.1% in the dexamethasone group), the sample size for this purpose was 29 cases in each of the two groups. The effect size of dexamethasone in relation to control was extrapolated to clonidine; therefore, the same sample size was established for this third group, but as the present study carried out a comparison among three groups, the final sample size of each group was expanded by 15% to correct for this difference. Thus, the sample size was set at 34 cases in each group to allow for a three-group comparison, considering a two-tailed significance level of 5% and a statistical power of 95%. The sample was increased by 15% to account for possible losses.

### Ethics statement

This study was approved by the Pontifical Catholic University of Paraná Institutional Review Board (CAAE: 33686720.8.1001.0020) and written informed consent was obtained from all subjects participating in the trial. The trial was registered prior to patient enrollment at https://ensaiosclinicos.gov.br/rg/ (RBR-48gkx3m), Principal investigator: Layana Vieira Nobre, Date of registration: February 25^th^, 2022). Patient enrollment occurred between March 1^st^, 2022 and December 31^th^, 2022.

### Statistical analysis

The data were compared among the three groups (Control, Dexamethasone, and Clonidine) using IBM SPSS Version 26.0. The Kolmogorov-Smirnov test was used to verify the assumption of normality. Categorical variables were compared using the chi-squared test, and numerical variables were compared using One-way Analysis of Variance (ANOVA) (parametric data) or Kruskal-Wallis test (nonparametric data), with post hoc analysis using the Bonferroni test; p-values < 0.05 were taken to indicate statistical significance.

## Results

**Figure 1:**
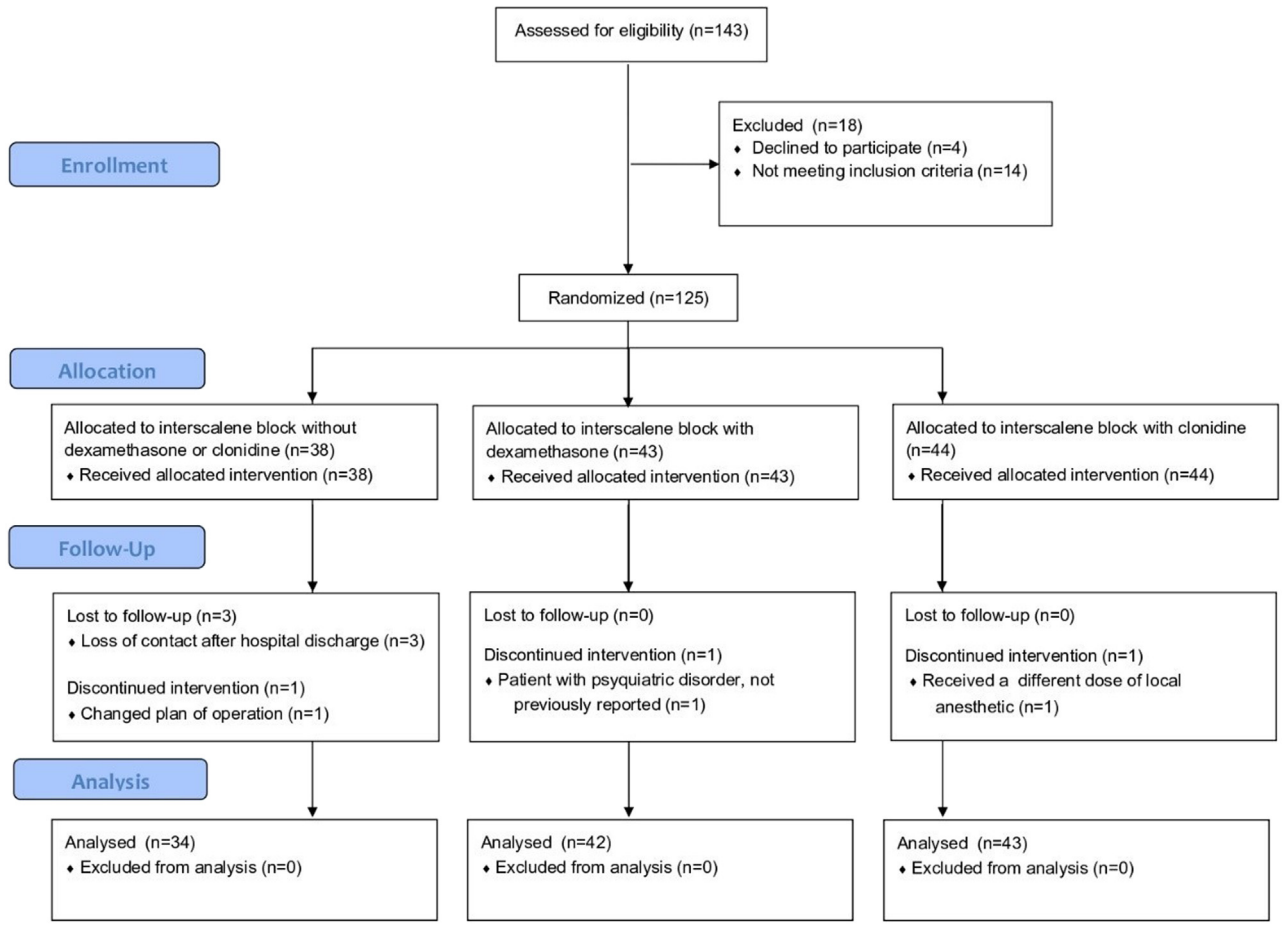
Consort flowchart.

A total of 119 patients were included for analysis: 34 in the control group, 42 in the dexamethasone group, and 43 in the clonidine group (Fig. 1).

Patients were similar in terms of sociodemographic data (Table 1).

**Table 1:** Sociodemographic data and surgical characteristics.

Variables	Control	Dexamethasone	Clonidine
Sample size, n	34	42	43
Sociodemography			
Age (years), Mean ± SD	50 ± 17	53 ± 15	51 ± 14
Gender, n (%)			
Male	22 (64.7)	28 (66.7)	28 (65.1)
Female	12 (35.3)	14 (33.3)	15 (34.9)
Anthropometry			
BMI (Kg.m^−2^), Mean ± SD	27 ± 4	28.3 ± 4.3	28.2 ± 4.5
Pre-surgical data			
Comorbidities, n (%)	17 (50.0)	24 (57.1)	22 (51.2)
ASA classification, n (%)			
I	17 (50.0)	18 (42.9)	21 (48.8)
II	17 (50.0)	24 (57.1)	22 (51.2)
Diagnosis, n (%)			
Fracture	11 (32.4)	15 (36.6)	15 (34.9)
AD	6 (17.6)	6 (14.6)	8 (18.6)
Rotator cuff tear	13 (38.2)	14 (34.1)	17 (39.5)
Others	4 (11.8)	6 (14.6)	3 (7.0)
Type of surgery, n (%)			
Open repair	19 (55.9)	23 (54.8)	19 (44.2)
Arthroscopy	15 (44.1)	19 (45.2)	24 (55.8)

ASA, American Society of Anesthesiologist; AD, Acromioclavicular Dislocation.

The incidence of rebound pain was 41.2% (95% CI 25.9‒57.9), 28.6% (95% CI 16.7‒43.3), and 23.3% (95% CI 12.6‒37.6) in the control, dexamethasone, and clonidine groups, respectively. There were no differences in the incidence of rebound pain among groups. There was no significant difference among the groups regarding the proportion of patients who reported pain after block resolution, and among the patients who reported such pain, there was no significant difference among the groups regarding the time (in hours) elapsed from block administration until the onset of pain. There was no significant difference among the groups regarding (i) The proportion of patients who received each analgesic regimen, (ii) The proportion of those who required rescue medication adverse events or complications, being observed in the clonidine group one case of bradycardia in the post-anesthesia recovery room (reversed with atropine) and another in the dexamethasone group, hypotension at the end of surgery (reversed with ephedrine), (iii) Or in the duration of rebound pain in hours (Table 2).

**Table 2:** Outcomes of interest across the three groups.

Variables	Control	Dexamethasone	Clonidine	p-value
Sample size, n	34	42	43	
Duration of surgery (min), Mean ± SD	165 ± 73	159 ± 73	149 ± 58	0.55^a^
Rebound pain, n (%)^e^	14 (41.2)	12 (28.6)	10 (23.3)	0.26^b^
Duration of rebound pain (hours), Median (Q_1_ – Q_3_)	2 (1 – 3)	3 (2 – 4)	3 (2 – 5)	0.08^c^
Complaint of pain after block, n (%)	27 (79.4)	36 (85.7)	32 (74.4)	0.42^b^
First complaint (hours) of pain after block, Median (Q_1_ – Q_3_)	12 (8 – 24)	12 (6 – 25)	12 (8 – 22)	0.78^c^
Satisfied, n (%)	33 (97.1)	40 (97.6)	43 (100.0)	–d
Postoperative non-opioid analgesics, n (%)	24 (70.6)	32 (76.2)	37 (86.0)	0.10^b^
Metamizole/Ketoprofen	13 (38.2)	15 (35.7)	26 (60.5)	
Metamizole	7 (20.6)	15 (35.7)	10 (23.3)	
Ketoprofen	4 (11.8)	2 (4.8)	1 (2.3)	
Rescue medications, n (%)	18 (52.9)	25 (59.5)	18 (41.9)	0.25^b^
Intraoperative complications, n (%)	0 (0.0)	1 (2.4)	0 (0.0)	–d
Postoperative complications, n (%)	0 (0.0)	0 (0.0)	1 (2.3)	–d

a One-way analysis of variance (ANOVA) parametric test.

b Chi-square test.

c Kruskal-Wallis nonparametric test. Median (Min‒Max): Median (Minimum‒Maximum).

d Conditions not met for the Chi-Square test; Satisfied: would you undergo interscalene block again?: yes/no. Postoperative non-opioid analgesics: number of patients who required each non-opioid analgesic. Rescue medications: number of patients who required tramadol or morphine even though they were taking non-opioid medications. SD, Standard Deviation.

e Simultaneously Sudden-onset rebound pain, VAS ≥ 7 and without improvement with oral medication; Q_1_: first quartile ‒ Q_2_: third quartile.

Comparison of the VAS-measured intensity of postoperative pain among the three groups also failed to show any significant difference at any time points evaluated and any significant difference in pain from baseline to each post-block time point (Table 3).

**Table 3:** Comparison of the three groups regarding pain outcomes scored at different time points (VAS) and the difference in pain from baseline to each post-block time point.

Outcome – Pain intensity (VAS)	Control	Dexamethasone	Clonidine	p-value^a^
Sample size, n	34	42	43	
Pain at rest, Median (Q_1_ ‒ Q_2_)				
Baseline (pre-block)	6 (3 | 8)	5 (4 | 7)	5 (2 | 7)	0.37
30-minutes after block	0 (0 | 0)	0 (0 | 1)	0 (0 | 0)	0.31
30-minutes in PACU	0 (0 | 0)	0 (0 | 0)	0 (0 | 0)	0.64
upon discharge from PACU	0 (0 | 0)	0 (0 | 1)	0 (0 | 0)	0.51
2 hours after discharge from PACU	0 (0 | 0)	0 (0 | 1)	0 (0 | 1)	0.32
4 hours after discharge from PACU	0 (0 | 0)	0 (0 | 3)	0 (0 | 2)	0.48
6 hours after discharge from PACU	0 (0 | 2)	0 (0 | 3)	0 (0 | 2)	0.53
12 hours after discharge from PACU	2 (0 | 5)	2 (0 | 3)	1 (0 | 5)	0.69
24 hours after discharge from PACU	2 (0 | 4)	3 (0 | 4)	2 (0 | 4)	0.47
48 hours after discharge from PACU	2 (0 | 3)	2 (0 | 3)	2 (0 | 3)	0.62
Difference in pain in each moment compared to baseline (pre-block) Median (Q_1_ ‒ Q_2_)				
30-minutes after blockade	-5 ([-8 | -3)	-4 (-7 | -3)	-4 (-7 | 0)	0.40
30-minutes in PACU	0 (0 | 0)	0 (0 | 0)	0 (0 | 0)	0.33
At discharge from PACU	0 (0 | 0)	0 (0 | 1)	0 (0 | 0)	0.46
2 hours after discharge from PACU	0 (0 | 0)	0 (0 | 0)	0 (0 | 0)	0.30
4 hours after discharge from PACU	0 (0 | 0)	0 (0 | 0)	0 (0 | 0)	0.13
6 hours after discharge from PACU	0 (0 | 0)	0 (0 | 0)	0 (0 | 0)	0.22
12 hours after discharge from PACU	0 (0 | 3)	0 (0 | 2)	0 (0 | 2)	0.27
24 hours after discharge from PACU	0 (|1 | 0)	0 (0 | 2)	0 (|2 | 1)	0.48
48 hours after discharge from PACU	0 (0 | 0)	0 (|1 | 0)	0 (|1 | 0)	0.98

PACU, Post-Anesthesia Care Unit; Q_1_, First quartile; Q_2_, Third quartile.

a Kruskal-Wallis nonparametric test.

## Discussion

In the present study, we did not find a reduction in rebound pain with either clonidine or dexamethasone. Our incidence of rebound pain (29.4%) was distinctly lower than previously reported in the literature (rates of up to 60%).^2^^,^^7^ Furthermore, there was no statistically significant between-group difference in postoperative analgesia with the current sample size.

This possibly occurred due to the study's conservative criteria of rebound pain. The strict definition of rebound pain used in this study, which considers only episodes of moderate to severe intensity (VAS ≥7), was intentionally adopted to ensure that only the most significant and impactful cases for the patient experience were included in the analysis. This choice aimed to provide a more precise assessment of relevant rebound pain in the clinical context, avoiding dilution of the results with less severe episodes that may not reflect the true burden of postoperative pain. Also, the discrepancy could be attributed to the postoperative analgesic regimen (metamizole and ketoprofen) used in the protocol. Conversely, many studies on rebound pain have not incorporated perioperative systemic analgesia, and outpatients generally receive less analgesia before discharge.^3^ Although there is no direct evidence that a consistent multimodal analgesic regimen reduces rebound pain, some studies recommend adequate perioperative analgesia associated with patient education on the duration of analgesia and on the use of oral analgesics before blockade resolution as a way to prevent rebound pain.^3^^,^^17^, ^18^, ^19^, ^20^, ^21^, ^22^ Therefore, the formulation of a perioperative care plan that includes around-the-clock analgesic coverage in the first 24 hours appears to be crucial.^19^

This discrepancy in the incidence of rebound pain with the literature can also be explained by the lack of consensus on how this phenomenon should be described or quantified. There is still no widely accepted method to quantify rebound pain, as researchers believe the exact timing of block resolution is challenging to pinpoint.^23^ In this study, we defined rebound pain concerning pain intensity and ancillary criteria described in several previous studies.^2^^,^^12^^,^^16^^,^^24^

It is important to emphasize that “rebound pain” and “postoperative pain” are distinct phenomena that require clear definitions. Rebound pain refers to a sudden increase in pain intensity, often associated with the end of an analgesic blockade, whereas postoperative pain refers to pain that may occur during the recovery period, regardless of the anesthetic technique used. This distinction is crucial for the interpretation of results and clinical application since management strategies and expectations regarding each type of pain can differ substantially.

The combination of peripheral blockade with a local anesthetic and adjuvants is recommended by several authors.^16^^,^^20^ In these studies, adjuvants were associated with reductions in the incidence of rebound pain and postoperative pain scores. However, in the present study, two separately studied single adjuvants, at least at the doses administered, did not significantly impact the incidence of rebound pain or postoperative pain. Other studies have also failed to find significant results, which shows a lack of consensus on a potential dose-effect relationship.^25^, ^26^, ^27^ These discrepancies may be associated with the broad variation in doses used across different studies, as there is also no consensus on the optimal dose of adjuvants, and also by the volume and concentration of local anesthetics. In another study, neither intravenous nor perineural dexamethasone at a dose of 4 mg prolonged the duration of ulnar nerve blockade.^28^ Moreover, another study demonstrated that only intravenous dexamethasone at a dose of 10 mg increased the duration of postoperative analgesia after sciatic nerve block compared to lower doses (2.5 mg or 5 mg) or no adjuvant. Furthermore, higher doses of perineural dexamethasone (4 mg vs. 2 mg) were associated with higher rebound pain scores.^28^^,^^29^

However, a meta-analysis with twenty-five studies showed significantly longer analgesic effects compared to the control group and, consequently, better postoperative pain scores with the use of perineural adjuvants (dexmedetomidine and dexamethasone).^10^ As mentioned before, methodological differences in the definition of rebound pain may explain this divergence from the present study's findings. The type of local anesthetic, doses, and concentrations were also different from those used in our study, which may have further impacted results.

Further studies are needed to elucidate the optimal dose of dexamethasone and other adjuvants that will be beneficial in reducing and possibly preventing rebound pain.

Our study makes a significant contribution to the understanding of rebound pain. One of the main strengths of our work is introducing a new definition of rebound pain, which enriches the understanding of this phenomenon. Furthermore, unlike previous studies, a multicenter study was carried out, which increases the generalization of the results. Finally, a multimodal postoperative pain control protocol was developed to reproduce in future studies.

The present study had limitations. We administered only one dose of local anesthetic, dexamethasone, and clonidine, and lacked a parallel control between different doses; the optimal dose has yet to be determined and established in the literature. Therefore, we cannot state conclusively that the lack of positive results was due to the choice of anesthetic and adjuvants, as these variables can play a determining role in the results.

Furthermore, the dose/concentration of local anesthetic used in the study is likely hyperalgesic.^14^ An idea for future studies, with the association of interscalene plexus block with general anesthesia, would be a lower concentration of local anesthetic such as 0.20%‒0.25% and letting the adjuvants possibly show their effects.

Added to this, a lower percentage of rebound pain was observed in the groups evaluated concerning that predicted in the sample calculation. This may have occurred due to the innovative classification used and the postoperative analgesic regimen. Therefore, new clinical trials that can evaluate possible factors that interfere with the occurrence of rebound pain and its prevention methods are essential.

However, the limitations mentioned above, such as the low incidence of rebound pain in the sample, may have compromised the study's statistical power since the calculations were based on high prevalence. New studies with larger sample sizes, meta-analyses incorporating underpowered studies, or using this study as an a priori probability for Bayesian analysis in a new trial may be potential solutions to address this problem.

We conclude that, at the doses used herein, administration of dexamethasone or clonidine as perineural adjuncts to local anesthesia in single-injection interscalene block did not significantly reduce the incidence of rebound pain compared to local anesthesia alone in patients undergoing shoulder surgery. Furthermore, there was no statistically significant between-group difference in postoperative analgesia.

## Clinical trial

This study was approved by the Pontifical Catholic University of Paraná Institutional Review Board (CAAE: 33686720.8.1001.0020) and written informed consent was obtained from all subjects participating in the trial. The trial was registered prior to patient enrollment at https://ensaiosclinicos.gov.br/rg/ (RBR-48gkx3m), Principal investigator: Layana Vieira Nobre, Date of registration: February 25^th^, 2022). The patient enrollment occurred between the March 1^st^, 2022 and December 31^th^, 2022.

## Conflicts of interest

The authors declare no conflicts of interest.
